# Can Blood Cell Count‐Based Inflammatory Markers Reflect the Risk of Osteoporosis? A Cross‐Sectional and Genetic Analysis

**DOI:** 10.1002/iid3.70449

**Published:** 2026-04-20

**Authors:** Jie Jin, Cheng Yu, Feng Chen, Jiajun Li, Yue Zhu, Yi Gao, Jingyi Wang, Feitian Ni, Ruotong Yao, Siyao Chen, Bohuai Yu, Yangguang Lu, Kai Huang, Kaiting Wu

**Affiliations:** ^1^ Department of Acupuncture and Moxibustion Tongde Hospital of Zhejiang Province Hangzhou China; ^2^ Department of Orthopedics Tongde Hospital of Zhejiang Province Hangzhou China; ^3^ The Second School of Medicine Wenzhou Medical University Wenzhou China; ^4^ School of Public Health Wenzhou Medical University Wenzhou China; ^5^ The Second Affiliated College Zhejiang Chinese Medical University Hangzhou China; ^6^ The First School of Medicine, School of Information and Engineering Wenzhou Medical University Wenzhou China; ^7^ Medical Comprehensive Ward, Xianlin Campus, Tongde Hospital of Zhejiang Province Hangzhou China

**Keywords:** blood cell count, cross‐sectional study, geroscience, inflammatory markers, Mendelian randomization, osteoporosis

## Abstract

**Background:**

Osteoporosis (OP) is characterized by reduced bone mineral density and bone structural deterioration, with a growing global prevalence. This study aims to explore the relationship between systemic inflammatory markers (SII, SIRI, AISI) and OP risk, and to assess their causal effects using genetic methods.

**Methods:**

A cross‐sectional analysis was conducted using data from 8,070 eligible participants. The relationships between SII, SIRI, AISI, bone mineral density, and OP risk were assessed through weighted multivariable regression and smooth curve fitting. Subgroup analyses examined the moderating effects of factors such as BMI and alcohol consumption. Mendelian randomization analysis was also performed using GWAS data to explore the causal relationship between blood cell counts and OP.

**Results:**

Elevated levels of SII, SIRI, and AISI were significantly associated with decreased bone mineral density and increased OP risk, with these associations remaining significant after adjusting for various confounders. Subgroup analyses revealed that the association between SII and OP was more pronounced in individuals with low BMI and those who consumed alcohol. Genetic analysis further provided evidence supporting that higher levels of specific blood cells, such as platelets and eosinophils, serve as causal factors contributing to increased OP risk.

**Conclusions:**

This study demonstrates that SII, SIRI, and AISI, as reliable indicators of systemic inflammation, can effectively predict OP risk, particularly in populations with low BMI and those who consume alcohol. These inflammatory markers may serve as tools for early OP screening and offer new insights for personalized prevention and intervention strategies.

## 1 Introduction

Osteoporosis (OP) is a significant global public health issue, characterized by decreased bone mineral density (BMD) and microstructural bone degradation. It is increasingly prevalent among women over 55 and men over 65 years of age [1, 2]. According to the International Osteoporosis Foundation, one in three women and one in five men over the age of 50 will experience an osteoporotic fracture in their lifetime [3]. Furthermore, OP leads to a range of bone‐related complications, increases mortality rates, and exacerbates the socio‐economic and healthcare burden [4]. Therefore, identifying new risk factors or early diagnostic biomarkers for OP is crucial for its prevention and treatment.

In recent years, the relationship between visceral adipose tissue, blood uric acid, serum aldosterone levels, and other common chronic conditions in middle‐aged and elderly individuals with OP and fracture outcomes has been further elucidated. Serum inflammatory‐immune markers associated with OP have gradually garnered attention as risk factors for OP [5, 6, 7]. Systemic inflammation and immune responses are closely linked to OP [8, 9]. The inflammatory immune microenvironment, composed of immune cells and inflammatory cytokines (such as IL‐1β, IL‐18, IL‐17, IL‐6, and TNF‐α), plays a key regulatory role in bone metabolism and contributes to the development of OP [10]. Previous studies have shown that the number of osteoclasts in the bone marrow of IL‐1 receptor knockout mice is significantly reduced, suggesting that the immune‐inflammatory microenvironment induced by IL‐1β may affect osteoclast‐mediated bone loss [11]. Inflammatory factors such as C‐reactive protein (CRP) and tumor necrosis factor‐α (TNF‐α) also act on mesenchymal stem cells and osteoclast precursors to enhance osteoclast‐mediated bone resorption [12, 13]. Additionally, the cascade of inflammatory cytokines and chemokines can ultimately lead to the aggregation of B lymphocytes and neutrophils, disrupting the balance between bone formation and resorption [14]. Therefore, inflammatory responses and related blood cells play an essential role in the pathogenesis of OP, warranting further investigation into the relationship between inflammatory biomarkers and OP.

Systemic immune‐inflammatory indices, such as the Systemic Immune‐Inflammation Index (SII), Systemic Inflammation Response Index (SIRI), and the Overall Inflammation Score (AISI), derived from blood cell counts, are widely regarded as reliable markers of systemic inflammation [15, 16, 17]. Among these, SII has been proposed as a predictor of low BMD or OP in postmenopausal women aged ≥ 50 years [18]. The platelet‐to‐lymphocyte ratio is negatively correlated with lumbar spine BMD and may serve as a potential inflammatory predictor for OP [19]. However, the relationship between peripheral inflammatory blood cell counts and OP from a genetic perspective remains underexplored. Therefore, in this study, we explore the association between SII, SIRI, AISI, and OP using large‐scale epidemiological databases. By employing Mendelian randomization (MR) methods, we investigate the role of genetic variants related to peripheral blood cell counts as instrumental variables (IVs) to reveal their genetic contribution to OP, offering new insights into its pathogenesis [20].

## 2 Materials and Methods

### 2.1 Study Design

We conducted a cross‐sectional study using data from the National Health and Nutrition Examination Survey (NHANES), an ongoing national survey that collects representative health data from the U.S. population every 2 years. Detailed study design, methods, and data collection are available on the NHANES website (https://www.cdc.gov/nchs/nhanes). Additionally, we adhered to the Strengthening the Reporting of Observational Studies in Epidemiology Using Mendelian Randomization guidelines [21]. Summary data from 29 genome‐wide association studies (GWAS) on blood cell counts were used to select appropriate single nucleotide polymorphisms (SNPs) as IVs for MR analysis, aiming to investigate causal relationships. As all data were derived from previously published studies and public databases, ethical approval was not required.

### 2.2 Cross‐Sectional Studies

#### 2.2.1 Study Participants

This study utilized NHANES data from the 2009–2018 cycles, excluding data from the 2011 to 2012 and 2015 to 2016 cycles due to the absence of BMD measurements. A total of 29,966 participants were initially included in this study. To ensure the representativeness and completeness of the research sample, we systematically screened participants according to the following criteria.

First, individuals younger than 20 years of age (*N*  =  12,410) were excluded because the DXA BMD measurements in NHANES primarily target adults. Including adolescents, who are still undergoing skeletal development, could introduce biological variability and potential statistical bias. Second, participants with missing core variables were excluded, including those lacking demographic information (*N*  =  1891), medical history data (*N*  =  5872), or key biochemical indicators such as serum calcium, phosphorus, and vitamin D (*N*  =  554). In addition, individuals without complete data on blood cell counts or femoral BMD (*N*  =  1169) were excluded.

**Figure 1:**
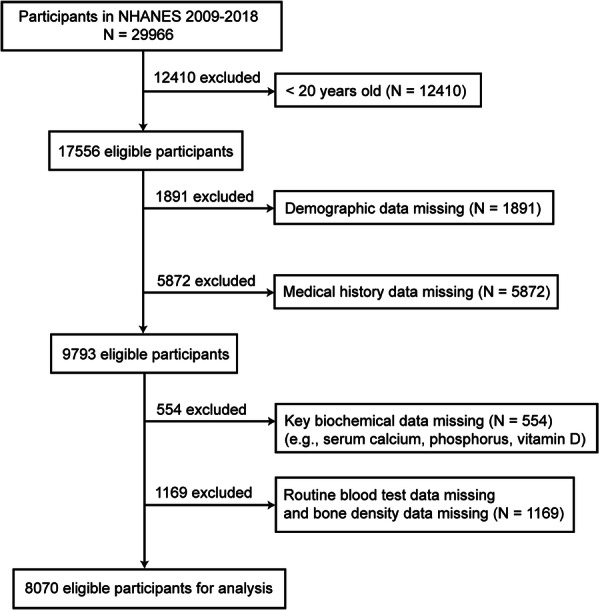
The flowchart of the participants' selection process. Participants under 20 years of age and those with missing demographic data, medical history, key biochemical indicators (e.g., serum calcium, phosphorus, and vitamin D), blood cell count data, or femoral bone mineral density (BMD) measurements were excluded. Eligible participants were defined as individuals who met all inclusion criteria and had complete data on demographic characteristics, clinical history, laboratory markers, and BMD. A total of 8,070 such participants were included in the final weighted analysis to ensure national representativeness.

After these rigorous exclusion steps, a total of 8,070 participants met all inclusion criteria and were defined as the “eligible participants” of this study. These participants had complete blood count parameters, bone density measurements, and demographic and clinical information, which allowed for the calculation of systemic inflammation indices (SII, SIRI, AISI) and the assessment of osteoporosis risk. Weighted analyses were performed according to the NHANES complex sampling design to ensure that the results were representative of the general adult population in the United States (Figure 1).

#### 2.2.2 Blood Cell Inflammation Indicators

Blood samples were analyzed using the Beckman Coulter HMX Hematology Analyzer, following the NHANES Laboratory Procedures Manual. We included three blood cell biomarkers related to systemic immune‐inflammatory responses [22, 23, 24]: SII = (absolute neutrophil count * platelet count)/absolute lymphocyte count, SIRI = (absolute neutrophil count * absolute monocyte count)/absolute lymphocyte count, and AISI = (absolute neutrophil count * platelet count * absolute monocyte count)/absolute lymphocyte count. Since these inflammatory biomarkers were skewed, we log‐transformed SII, SIRI, and AISI using log10 for regression analysis to improve statistical power.

#### 2.2.3 Bone Density and OP

BMD was measured using a Hologic QDR‐4500A fan‐beam densitometer (Hologic Inc., Bedford, MA, USA). OP was defined according to World Health Organization criteria as a BMD value below −2.5 standard deviations from the young adult reference population at any femoral site [25]. The femoral regions assessed included the total femur, femoral neck, trochanter, and intertrochanter.

#### 2.2.4 Covariates

We considered additional covariates that may affect bone metabolism, based on previous studies and clinical guidelines [18, 26]. NHANES researchers collected demographic information, including sex, age, race, education level, poverty income ratio (PIR), and body mass index(BMI). Medical history data included alcohol use, smoking, diabetes, family history of OP, milk consumption, and physical activity. Other laboratory tests included alanine aminotransferase (ALT), aspartate aminotransferase (AST), blood calcium, phosphorus, serum creatinine, and serum 25‐hydroxyvitamin D.

#### 2.2.5 Statistic Analysis

Data are presented as mean ± standard deviation (SD). Statistical significance between two groups was assessed using the Student's *t*‐test, while comparisons among three or more groups were performed using one‐way analysis of variance (ANOVA). Weighted multivariable linear regression was used to assess the relationship between each inflammatory biomarker and BMD. Weighted multivariable logistic regression was employed to evaluate the association between inflammatory biomarkers and OP. The generalized additive model (GAM) and smooth curve fitting were used to visualize the relationship between inflammatory biomarkers and BMD, and subgroup analyses were performed using stratified logistic regression models. Interaction effects among subgroups were assessed using likelihood ratio tests. All statistical analyses were conducted using R 4.3.2. Differences were considered statistically significant at a level of *p* < 0.05, with results indicated as follows: *p < 0.05, **p < 0.01, ***p < 0.001.

### 2.3 Mendelian Randomization

#### 2.3.1 GWAS Data Sources

The GWAS data for 29 blood cell counts were obtained from a 2020 study by Dr. Dragana Vuckovic and colleagues, which included 563,085 individuals of European descent [27]. Summary statistics for each blood cell count GWAS are publicly available in the GWAS catalog (accession numbers GCST90002379 to GCST90002407). Genetic data on OP were sourced from the FinnGen consortium (https://r10.finngen.fi/), which included 8,017 OP cases and 391,037 healthy controls. All participants were of European ancestry and provided informed consent. Case identification was based on the International Classification of Diseases, 10th Edition (ICD‐10) codes. Detailed participant characteristics, genotyping, imputation, and quality control methods are available on the FinnGen website (https://finngen.gitbook). All DNA positions were based on the human reference genome hg19 (GRCh37). There is no overlap between the study populations in the different GWAS datasets, making them suitable for MR analysis.

#### 2.3.2 Instrumental Variable Selection

To ensure the robustness of the MR analysis, we applied the following criteria for selecting instrumental variables (IVs). SNPs were selected if they were associated with the exposure at significance thresholds of 5 × 10⁻⁸ and 1 × 10⁻⁵. SNPs with strong linkage disequilibrium (LD) were excluded, as strong LD can introduce bias (*r*² < 0.001, clumping distance = 10,000 kb). In cases of LD, the variant with the lowest *p*‐value for the exposure was selected. Weak instruments (F < 10) were also excluded to ensure a strong correlation between the IVs and the exposure. Finally, we harmonized the SNPs for both exposure and outcome to ensure consistent effect estimates for the same allele, excluding palindromic SNPs with intermediate allele frequencies or incompatible alleles.

#### 2.3.3 Statistical Analysis

We used the inverse variance weighted (IVW) method to assess the correlation between the exposure and outcome. When all IVs satisfy the three key assumptions, the IVW method provides accurate and stable estimates [28]. Results are presented as odds ratios (ORs) with 95% confidence intervals (CIs). Cochran's Q test was used to assess heterogeneity, quantified by the I² statistic. A low I² value (< 25%) indicates no heterogeneity, while *I*² < 50% suggests mild heterogeneity. We assessed potential horizontal pleiotropy using the intercept from MR‐Egger regression [29, 30]. To ensure result robustness, sensitivity analyses were performed using additional MR models, including MR‐Egger, Weighted Median, and Simple Median methods. Statistical significance was defined as *p* < 0.05, with results indicated as follows: *p < 0.05, **p < 0.01, ***p < 0.001. MR analyses were conducted using R version 4.3.2.

## 3 Results

### 3.1 Baseline Characteristics of Population Divided by OP

The baseline characteristics of the population, categorized by OP, are shown in Table 1. Among the 8,070 participants, 445 had OP. Compared to individuals without OP, those with OP were older, predominantly female, non‐Hispanic white, had lower education levels, lower income‐to‐poverty ratios, and lower BMI. They were also more likely to drink alcohol, had a family history of OP, and exhibited differences in physical activity and various biomarkers, including alanine transaminase, aspartate transaminase, blood phosphate, serum creatinine, serum 25‐hydroxyvitamin D, lymphocyte count, monocyte count, SII, SIRI, and AISI (*p* < 0.05).

**Table 1:** Baseline characteristics of populations classified by osteoporosis status.

Variables	Total (*N* = 8070)	Osteoporosis (*N* = 445)		
		Yes	No	*p*‐value
Age, years	55.13 ± 15.88	69.86 ± 11.83	54.27 ± 15.67	< 0.001***
Male, %	51.19	33.03	52.25	< 0.001***
Race, %				< 0.001***
Mexican American	15.09	7.64	15.53	
Other Hispanic	9.68	6.74	9.85	
Non‐Hispanic White	47.32	64.72	46.31	
Non‐Hispanic Black	18.28	8.09	18.87	
Other races	9.63	12.81	9.44	
Education level, %				< 0.001***
< high school	23.20	28.54	22.89	
High school	23.49	26.74	23.30	
> high school	53.31	44.72	53.81	
PIR	2.24 (1.16, 4.27)	1.94 (1.11, 3.40)	2.27 (1.17, 4.35)	< 0.001***
BMI, kg/m2	28.70 ± 5.94	24.94 ± 4.94	28.92 ± 5.92	< 0.001***
Diabetes, %	17.72	19.10	17.64	0.616
Drinking, %	77.45	65.84	78.12	< 0.001***
Smoking, %	47.01	46.29	47.06	0.098
Family history of osteoporosis, %	11.77	17.75	11.42	< 0.001***
Physical activity, %	36.41	21.57	37.27	< 0.001***
Milk product consumption, %	82.48	80.00	82.62	0.157
ALT, U/L	20.00 (16.00, 28.00)	16.00 (13.00, 21.00)	21.00 (16.00, 28.00)	< 0.001***
AST, U/L	23.00 (19.00, 27.00)	22.00 (19.00, 26.00)	23.00 (19.00, 27.00)	0.012*
Serum creatinine, mg/dL	0.88 (0.74, 1.03)	0.86 (0.72, 1.06)	0.88 (0.74, 1.03)	0.024*
Serum calcium, mg/dL	9.40 (9.20, 9.60)	9.40 (9.20, 9.60)	9.40 (9.10, 9.60)	0.401
Serum phosphorus, mg/dL	3.70 (3.30, 4.10)	3.80 (3.40, 4.10)	3.70 (3.30, 4.10)	0.002**
Serum 25(OH)D, nmol/L	66.00 (49.00, 85.30)	77.10 (53.80, 96.40)	65.50 (48.80, 84.70)	< 0.001***
Lymphocyte, 109/L	2.00 (1.60, 2.50)	1.80 (1.40, 2.40)	2.00 (1.60, 2.50)	< 0.001***
Monocyte, 109/L	0.50 (0.40, 0.70)	0.60 (0.40, 0.70)	0.50 (0.40, 0.70)	0.005**
Neutrophil, 109/L	3.90 (3.10, 5.00)	4.00 (3.30, 5.00)	3.90 (3.10, 5.00)	0.577
Platelet, 109/L	228.00 (193.00, 270.00)	219.00 (185.00, 269.00)	229.00 (194.00, 270.00)	0.363
SII	444.46 (317.34, 624.67)	481.58 (329.12, 686.11)	442.50 (317.03, 621.00)	< 0.001***
SIRI	1.04 (0.71, 1.54)	1.16 (0.82, 1.85)	1.03 (0.70, 1.52)	< 0.001***
AISI	235.84 (152.03, 366.89)	262.97 (172.67, 414.10)	233.83 (151.31, 363.73)	< 0.001***

Abbreviations: AISI, aggregate index of systemic inflammation; BMI, body mass index; PIR, poverty‐income ratio; SII, systemic immune‐inflammation index; SIRI, systemic inflammation response index. Differences are indicated as follows: *p < 0.05, **p < 0.01, ***p < 0.001.

### 3.2 Multi‐Factor Linear Regression and Logistic Regression

**Figure 2:**
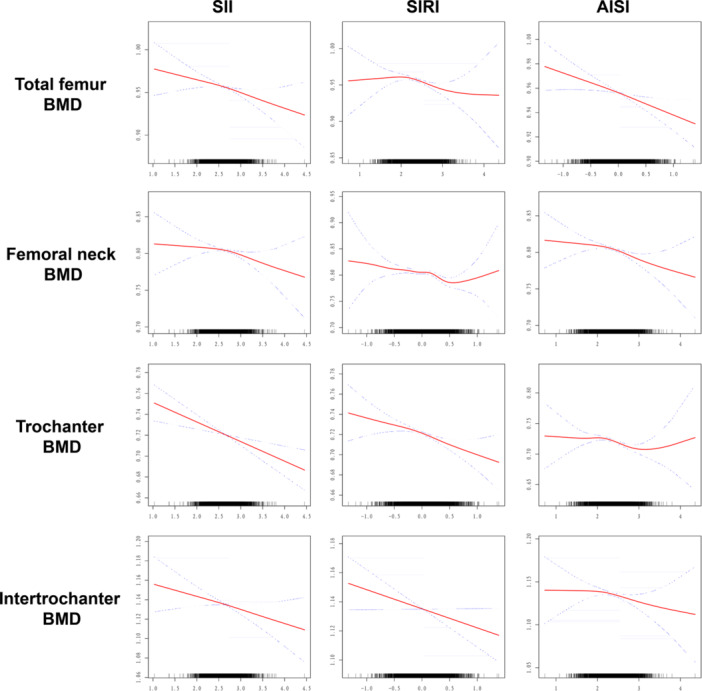
Smooth curve fitting showing the relationship between inflammatory biomarkers and bone mineral density. AISI, aggregate index of systemic inflammation; BMI, body mass index; SII, systemic immune‐inflammation index; SIRI, systemic inflammation response index.

We used weighted multivariable linear regression to explore the relationship between inflammatory markers and BMD, and weighted multivariable logistic regression to assess the association between inflammatory markers and OP. Two models were employed (Table 2): Model 1 adjusted for sex and age (grouped by age 60, differentiating elderly and non‐elderly groups); Model 2 was fully adjusted for all covariates. In Model 1, higher log‐transformed SII, SIRI, and AISI were associated with lower BMD at various sites and increased OP risk (*p* < 0.05). In Model 2, each unit increase in log‐transformed SII was associated with a decrease in femoral total BMD by 0.016 units (95% CI: −0.028 to −0.004), femoral neck BMD by 0.013 units (95% CI: −0.025 to −0.001), and trochanteric BMD by 0.019 units (95% CI: −0.029 to −0.008). Each unit increase in log‐transformed SIRI was associated with a decrease in femoral total BMD by 0.018 units (95% CI: −0.029 to −0.006), femoral neck BMD by 0.022 units (95% CI: −0.033 to −0.011), and trochanteric BMD by 0.020 units (95% CI: −0.030 to −0.010). OP risk increased by 2.019 times (95% CI: 1.346, 3.030). For AISI, each unit increase was associated with a decrease in femoral total BMD by 0.015 units (95% CI: −0.024 to −0.005), femoral neck BMD by 0.017 units (95% CI: −0.026 to −0.007), and trochanteric BMD by 0.017 units (95% CI: −0.026 to −0.009). OP risk increased by 1.645 times (95% CI: 1.162 to 2.331). Smoothing curve fitting showed a linear dose‐response relationship between log‐transformed SII, SIRI, AISI, and BMD at various sites (*P* for linearity < 0.001), suggesting that higher inflammation levels are closely associated with decreased BMD (Figure 2).

**Table 2:** Multivariate regression model evaluating the relationship between inflammatory biomarkers, bone mineral density, and osteoporosis.

Classifications			SII	SIRI	AISI
Total femur BMD	Model 1	*β* (95% CI)	−0.025 (−0.038, −0.011)	−0.028 (−0.040, −0.016)	−0.016 (−0.027, −0.006)
		*p*‐value	< 0.001***	< 0.001***	0.002***
	Model 2	*β* (95% CI)	−0.016 (−0.028, −0.004)	−0.018 (−0.029, −0.006)	−0.015 (−0.024, −0.005)
		*p*‐value	0.010**	0.002**	0.003**
Femoral neck BMD	Model 1	*β* (95% CI)	−0.030 (−0.043, −0.017)	−0.041 (−0.052, −0.029)	−0.025 (−0.036, −0.015)
		*p*‐value	< 0.001***	< 0.001***	< 0.001***
	Model 2	*β* (95% CI)	−0.013 (−0.025, −0.001)	−0.022 (−0.033, −0.011)	−0.017 (−0.026, −0.007)
		*p*‐value	0.032*	< 0.001***	< 0.001***
Trochanter BMD	Model 1	*β* (95% CI)	−0.024 (−0.035, −0.012)	−0.025 (−0.035, −0.015)	−0.017 (−0.026, −0.008)
		*p*‐value	< 0.001***	< 0.001***	< 0.001***
	Model 2	*β* (95% CI)	−0.019 (−0.029, −0.008)	−0.020 (−0.030, −0.010)	−0.017 (−0.026, −0.009)
		*p*‐value	< 0.001***	< 0.001***	< 0.001***
Intertrochanter BMD	Model 1	*β* (95% CI)	−0.024 (−0.040, −0.008)	−0.025 (−0.040, −0.011)	−0.013 (−0.026, −0.000)
		*p*‐value	0.003**	< 0.001***	0.042*
	Model 2	*β* (95% CI)	−0.014 (−0.028, 0.001)	−0.013 (−0.027, 0.000)	−0.011 (−0.023, 0.001)
		*p*‐value	0.065	0.056	0.065
Osteoporosis	Model 1	OR (95% CI)	2.048 (1.364, 3.075)	2.698 (1.860, 3.915)	1.952 (1.410, 2.701)
		*p*‐value	< 0.001***	< 0.001***	< 0.001***
	Model 2	OR (95% CI)	1.506 (0.979, 2.316)	2.019 (1.346, 3.030)	1.645 (1.162, 2.331)
		*p*‐value	0.062	< 0.001***	0.005**

Abbreviations: AISI, aggregate index of systemic inflammation; BMI, body mass index; PIR, poverty‐income ratio; SII, systemic immune‐inflammation index; SIRI, systemic inflammation response index. Differences are indicated as follows: *p < 0.05, **p < 0.01, ***p < 0.001.

### 3.3 Subgroup Analysis

**Figure 3:**
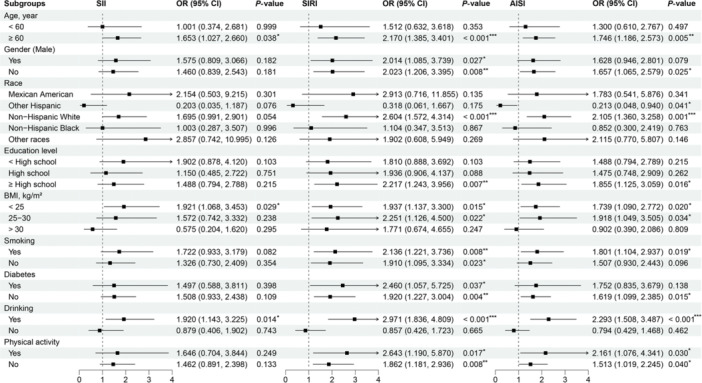
Subgroup analysis of the relationship between inflammatory biomarkers and osteoporosis. AISI, aggregate index of systemic inflammation; BMI, body mass index; SII, systemic immune‐inflammation index; SIRI, systemic inflammation response index. Differences are indicated as follows: *p < 0.05, **p < 0.01, ***p < 0.001.

Subgroup analyses were conducted with covariates such as age, sex, race, education, BMI, smoking, diabetes, alcohol consumption, and physical activity. Results showed that log‐transformed SII remained associated with OP in subgroups aged 60 or older, with BMI < 25 kg/m², and those who consumed alcohol. Log‐transformed SIRI remained associated with OP in subgroups aged 60 or older, male, non‐Hispanic white, with a high school education or higher, BMI < 30 kg/m², and those who smoked, drank alcohol, had diabetes, or engaged in physical activity. Log‐transformed AISI remained associated with OP in subgroups aged 60 or older, female, non‐Hispanic white, other Hispanic, with a high school education or higher, BMI < 30 kg/m², and those who smoked, drank alcohol, did not have diabetes, or engaged in physical activity (Figure 3). Interaction analysis revealed a significant interaction between alcohol consumption and inflammatory markers (*p* = 0.002), warranting further investigation into their mechanistic relationship.

### 3.4 Evidence From Mendelian Randomization

**Figure 4:**
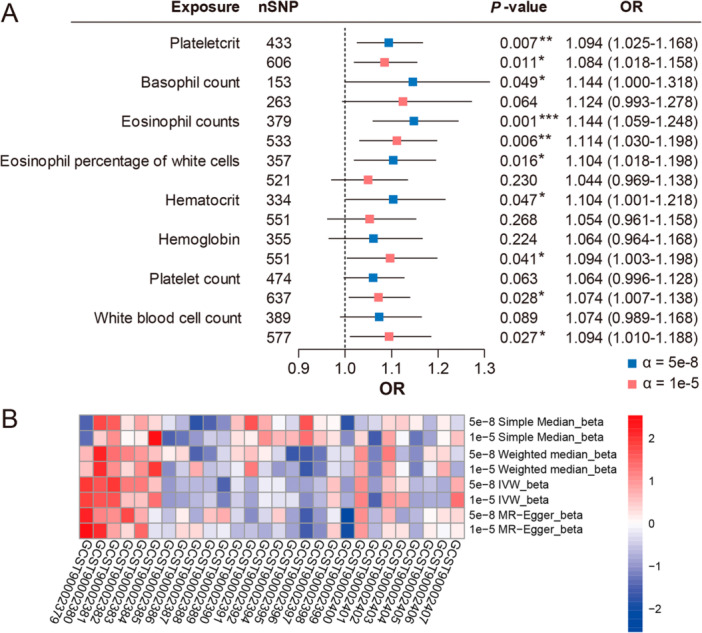
Results of genetic analyses carried out using Mendelian randomization methods. (A) Positive causal associations between blood cells and osteoporosis obtained at significance levels of α = 5e‐8 or α = 1e‐5, respectively, assessed using the IVW model; Differences are indicated as follows: *p < 0.05, **p < 0.01, ***p < 0.001. (B) Heatmap of the results of sensitivity analyses carried out by calling multiple Mendelian randomization models.

At a significance threshold of α = 5e‐8, MR analysis using the IVW model showed statistically significant associations between increased platelet hematocrit (*p* = 0.007, OR = 1.094, 95% CI: 1.025–1.168), basophil count (*p* = 0.049, OR = 1.144, 95% CI: 1.000–1.318), eosinophil count (*p* = 0.001, OR = 1.144, 95% CI: 1.059–1.248), eosinophil proportion in white blood cells (*p* = 0.016, OR = 1.104, 95% CI: 1.018–1.198), and increased hematocrit (*p* = 0.047, OR = 1.104, 95% CI: 1.001–1.218) with elevated OP risk. Using a more relaxed IV significance threshold of α = 1e‐5, associations between increased platelet hematocrit (*p* = 0.017, OR = 1.084, 95% CI: 1.018–1.158) and eosinophil count (*p* = 0.006, OR = 1.114, 95% CI: 1.030–1.198) with OP remained significant (Data S1–S2). Additionally, a significant causal relationship was observed with increased hemoglobin (*p* = 0.041, OR = 1.094, 95% CI: 1.003–1.198), platelet count (*p* = 0.028, OR = 1.074, 95% CI: 1.007–1.138), and white blood cell count (*p* = 0.027, OR = 1.094, 95% CI: 1.010–1.188) as risk factors for OP (Figure 4A). These results align with cross‐sectional studies regarding direction and effect. Despite differences in significance and effect size across various IV thresholds, the direction of association remained consistent.

Sensitivity analyses using MR‐Egger and other MR methods showed similar effect sizes and directions across models (Figure 4B). Cochran's Q test for heterogeneity revealed significant heterogeneity among instrumental variables for each exposure (*p* < 0.05), but mild heterogeneity was observed for eosinophil count, basophil count, hematocrit, and hemoglobin (25% ≤ *I*² < 50%). No evidence of horizontal pleiotropy was found in the MR‐Egger intercept test (*p* > 0.05) (Table 3). Thus, the MR analysis results are robust.

**Table 3:** Heterogeneity and horizontal pleiotropy of positive results of Mendelian randomization analysis with blood cell count as exposure (based on α = 5e‐8) and osteoporosis as outcome.

Trails	Heterogeneity		Pleiotropy		
	*I* ^2^	*p* value	EI	SE	*p* value
Plateletcrit	14.01%	0.011*	−0.002	0.002	0.309
Basophil count	26.27%	0.002**	−0.003	0.004	0.381
Eosinophil counts	26.21%	< 0.001***	0.000	0.002	0.961
Eosinophil percentage of white cells	22.49%	< 0.001***	−0.001	0.004	0.819
Hematocrit	25.00%	< 0.001***	−0.000	0.002	0.959
Hemoglobin	26.13%	< 0.001***	0.001	0.002	0.648
Platelet count	16.89%	0.002**	−0.002	0.002	0.921
White blood cell count	16.45%	0.005**	−0.000	0.002	0.975

Abbreviations: EI, Egger intercept; SE, standard error. Differences are indicated as follows: *p < 0.05, **p < 0.01, ***p < 0.001.

## 4 Discussion

This study is the first to systematically assess the relationship between systemic inflammatory markers, BMD, and the risk of OP. The cross‐sectional analysis revealed significant associations between elevated SII, SIRI, and AISI with decreased BMD and increased OP risk. Our MR analysis provided genetic evidence supporting a causal relationship between blood cell traits and osteoporosis. The analysis demonstrated that genetically predicted higher levels of specific blood cells, such as platelets and eosinophils, act as causal factors contributing to increased osteoporosis risk. These findings are highly consistent with and mutually reinforcing to our cross‐sectional results. These results underscore the critical role of inflammatory markers in the pathophysiology of OP.

The cross‐sectional analysis suggests that white blood cells, particularly neutrophils and monocytes, are key regulators of bone metabolism. Activation of these cells is often accompanied by the excessive secretion of pro‐inflammatory cytokines such as IL‐1β, TNF‐α, and IL‐6, which promote osteoclast differentiation and activity through the RANK/RANKL/OPG signaling pathway [31]. In parallel, eosinophils have been identified as active regulators of bone homeostasis, localizing near osteoclasts and restraining osteoclast differentiation and resorptive activity via eosinophil peroxidase in translational models [32], with recent commentaries outlining their emerging roles in skeletal regulation [33]. On the thrombopoietic side, megakaryocyte–bone cell interactions can stimulate osteoblast proliferation while constraining osteoclastogenesis, providing a mechanistic bridge from platelet biology to skeletal homeostasis [34, 35]. These convergent osteoimmune pathways offer a rationale for the observed associations between CBC‐derived composite indices and osteoporosis risk in our study. Specifically, IL‐1β stimulates osteoclast formation while inhibiting osteoblast differentiation, leading to increased bone resorption. TNF‐α directly induces bone matrix degradation by binding to osteoclast surface receptors. Genetic evidence also points to eosinophils and basophils playing a synergistic role in the inflammatory microenvironment. Eosinophils, in particular, were significantly associated with increased OP risk at both α = 5e‐8 and α = 1e‐5 significance levels. Their secretion of IL‐4 indirectly regulates osteoblast differentiation while promoting matrix metalloproteinase (MMP) activity, which accelerates bone matrix degradation [32]. Additionally, basophils release histamine, increasing local vascular permeability and possibly enhancing osteoclast activity by modulating the bone marrow microenvironment [36]. These inflammatory responses can lead to an imbalance between osteogenesis and osteoclastogenesis, accelerating bone loss and increasing OP risk.

Platelets, integral to SII, SIRI, and AISI, reflect changes in both inflammatory status and bone metabolism. An elevated platelet count often signals systemic inflammation. In the inflammatory microenvironment, platelets secrete growth factors such as TGF‐β and PDGF, which induce osteoclast precursor differentiation [37]. TGF‐β not only promotes osteoclast bone resorption but also inhibits osteoblast differentiation, exacerbating the bone metabolism imbalance. PDGF regulates the migration and proliferation of osteoblasts and fibroblasts during bone tissue repair [38]. MR analysis confirmed that increased platelet volume (MPV) was significantly associated with OP at both α = 5e‐8 and α = 1e‐5 thresholds, suggesting that platelets in an inflammatory state may intensify pathological processes. Activated platelets release pro‐inflammatory cytokines, form microthrombi, and disrupt bone microcirculation, leading to local hypoxia [39]. This suppresses osteoblast activity while promoting bone resorption, indicating that changes in platelet function and count may be crucial in the inflammatory modulation of OP.

A 2023 cross‐sectional study by Liu et al. [40], based on a Chinese population, found a positive correlation between hemoglobin levels and BMD, suggesting that low hemoglobin may be an independent risk factor for OP. Anemia could affect bone tissue through iron metabolism regulation, as iron overload can increase osteoclast activity via oxidative stress. Our genetic analysis at α = 1e‐5 revealed elevated hemoglobin as a risk factor for OP, which contrasts with previous studies. Integrating these findings with our inflammatory hypothesis, elevated hemoglobin may reflect blood concentration or a chronic hypoxic state, both of which can negatively affect bone metabolism. Increased blood viscosity may cause microcirculation issues in trabecular bone, leading to a hypoxic environment that enhances osteoclast activity and inhibits osteoblast differentiation via hypoxia‐inducible factor‐1α (HIF‐1α), contributing to OP [41, 42]. Elevated hemoglobin may also signal increased iron load, which promotes oxidative stress, damaging the bone matrix and stimulating osteoclast differentiation [43]. Iron overload inhibits key factors involved in bone formation, such as BMP‐2 and Runx2, accelerating bone loss [44]. Therefore, high hemoglobin levels may serve as an indirect marker of these pathological processes.

The findings of this study are consistent with previous research on the relationship between inflammation and OP, while also offering new insights and additional evidence. Recent reviews emphasize that chronic low‐grade inflammation and oxidative stress jointly disrupt bone remodeling and accelerate osteoclast‐mediated bone loss [45]. Contemporary population studies further associate CBC‐derived composite indices with lower BMD, osteoporosis, and even future fractures, supporting their biological and clinical plausibility for early risk triage [18, 46]. For example, a 2022 study by Tang et al. [18] suggested that SII could predict the risk of BMD decline in postmenopausal women. Our research not only confirms this conclusion but also expands the understanding of how inflammatory markers relate to BMD at various sites and OP risk. This is achieved through multivariate‐adjusted models and genetic causal analysis. Additionally, we explored how demographic and lifestyle factors influence the relationship between inflammatory markers and OP through subgroup analyses. For instance, we found that SII had a stronger association with OP in populations with low BMI or alcohol consumption, suggesting that chronic inflammation has a more pronounced effect in individuals with poor nutritional status. This finding extends previous literature and highlights the need for targeted interventions in high‐risk subgroups. Clinicians should pay particular attention to the nutritional status and inflammatory levels of patients with low BMI, using composite inflammatory indicators like SII for early screening and risk assessment. Nutritional interventions, such as increasing protein and vitamin D intake, may help improve bone metabolism and reduce OP risk. For individuals who consume alcohol, it is recommended to monitor and intervene in their drinking habits, encouraging reduced alcohol intake to lower chronic inflammation. Regular assessments of BMD and inflammation‐related markers should be performed to track disease progression. For patients diagnosed with or at high risk for OP, monitoring changes in SII and other markers during follow‐up, along with personalized anti‐inflammatory and nutritional support strategies, may optimize bone health management. This multidimensional approach could help improve OP outcomes in high‐risk populations.

We offered significant advantages and innovations in the field of OP etiology. First, by combining cross‐sectional data from large epidemiological databases with MR analysis, we provide a comprehensive understanding of the relationship between systemic inflammatory markers and OP risk from both epidemiological and genetic perspectives. This approach mitigates confounding bias that may arise in single observational studies, enhancing the reliability of causal inferences. Second, unlike previous studies that focused on single inflammatory factors, we innovatively assessed the relationship between composite inflammatory markers, based on blood cell counts, and BMD and OP. These markers reflect the overall burden of systemic immune‐inflammatory status, rather than the isolated effects of a single factor. Furthermore, by linking inflammatory markers with specific genetic features, such as platelet count and hemoglobin levels, we elucidate potential biological mechanisms, offering valuable insights for future basic research.

Despite its important findings, this study has several limitations. First, the cross‐sectional analysis utilized data from the NHANES database, which predominantly represents the U.S. population, while the MR analysis used GWAS data from individuals of European descent. This may limit the racial applicability and generalizability of the results. Second, while MR analysis offers strong causal inference, the validity of its results depends on the reliability of IVs. Although we applied multiple MR models and observed no significant horizontal pleiotropy among IVs, some genetic variations may still be subject to unrecognized bias. Additionally, although this study confirms significant and robust associations between SII, SIRI, AISI and reduced bone mineral density as well as increased osteoporosis risk, their clinical implications should be interpreted cautiously. These blood cell‐based inflammatory markers lack disease specificity, as elevated levels are observed in various systemic disorders. Therefore, they are not suitable as standalone diagnostic biomarkers for OP, but should rather be considered as risk prediction and screening tools. Finally, the cross‐sectional design does not account for dynamic changes in inflammatory markers, which may play different roles in the progression of OP.

This study provides new perspectives for early screening and personalized interventions for OP. Inflammatory composite markers, such as SII and SIRI, are cost‐effective and easily accessible screening tools with potential for identifying high‐risk populations and supporting early preventive strategies. Moreover, the clinical value of routine blood markers, such as platelet count and hemoglobin, may extend beyond their traditional uses, warranting further attention to their role in bone metabolism. Future research should validate these findings in multi‐ethnic and multi‐center populations, explore the dynamic relationship between inflammatory markers and bone metabolism, and clarify the mechanisms of key inflammatory factors and signaling pathways through molecular biological experiments. Additionally, future studies should investigate whether interventions targeting systemic inflammation can improve BMD or reduce fracture risk. By integrating basic research with clinical practice, new strategies for precise prevention and treatment of OP may be developed.

## 5 Conclusion

This study demonstrated that systemic inflammation markers (SII, SIRI, AISI) are significantly associated with reduced bone mineral density and increased OP risk, with robust findings supported by both epidemiological and genetic analyses. In addition, MR analysis pointed to the role of platelets and eosinophils in the development of OP. Subgroup analyses revealed stronger associations in individuals with lower BMI and alcohol consumption, highlighting chronic inflammation's role in vulnerable populations. Importantly, these indices are not proposed as standalone diagnostic or prognostic markers. Rather, they offer a pragmatic first‐line triage signal derived from routine blood counts, guiding targeted evaluation and prevention while avoiding over‐reliance on any single biomarker. These findings suggest that inflammation markers could serve as practical tools for early OP risk assessment and guide targeted prevention strategies, paving the way for more personalized approaches in OP care.

## Author Contributions

**Jie Jin:** methodology, validation, formal analysis, investigation, data curation, visualization, writing – original draft preparation, writing – review and editing. **Cheng Yu:** methodology, validation, formal analysis, data curation, visualization, writing – original draft preparation, writing – review and editing. **Feng Chen:** formal analysis, data curation, writing – review and editing. **Jiajun Li:** formal analysis, data curation, writing – review and editing. **Yue Zhu:** formal analysis, data curation, writing – review and editing. **Yi Gao:** formal analysis, data curation, writing – review and editing. **Jingyi Wang:** data curation, writing – review and editing. **Feitian Ni:** data curation, writing – review and editing. **Ruotong Yao:** data curation, writing – review and editing. **Siyao Chen:** writing – review and editing. **Bohuai Yu:** data curation, writing – review and editing. **Yangguang Lu:** conceptualization, investigation, resources, supervision, project administration, writing – original draft preparation, writing – review and editing. **Kai Huang:** conceptualization, investigation, resources, supervision, project administration, funding acquisition, writing – review and editing. **Kaiting Wu:** conceptualization, investigation, resources, supervision, project administration, writing – review and editing.

## Ethics Statement

The authors have nothing to report.

## Conflicts of Interest

The authors declare no conflicts of interest.

## Supporting information

Supporting File 1

Supporting File 2

## Data Availability

The data that support the findings of this study are openly available. Cross‐sectional population data were obtained from the National Health and Nutrition Examination Survey (NHANES) database(https://www.cdc.gov/nchs/nhanes). GWAS data for osteomyelitis were obtained from IEU Open GWAS Project (https://gwas.mrcieu.ac.uk/datasets/) and FinnGen consortium (https://r10.finngen.fi/).

## References

[1] J. E. Compston , M. R. McClung , and W. D. Leslie , “Osteoporosis,” Lancet 393, no. 10169 (2019): 364–376, 10.1016/s0140-6736(18)32112-3. 30696576

[2] B. Kirk , J. Zanker , and G. Duque , “Osteosarcopenia: Epidemiology, Diagnosis, and Treatment‐Facts and Numbers,” Journal of Cachexia, Sarcopenia and Muscle 11, no. 3 (2020): 609–618, 10.1002/jcsm.12567. 32202056 PMC7296259

[3] T. Sözen , L. Özışık , and N. Çalık Başaran , “An Overview and Management of Osteoporosis,” European Journal of Rheumatology 4, no. 1 (2019): 46–56, 10.5152/eurjrheum.2016.048. PMC5335887 28293453

[4] T. D. Rachner , S. Khosla , and L. C. Hofbauer , “Osteoporosis: Now and the Future,” Lancet 377, no. 9773 (2011): 1276–1287, 10.1016/s0140-6736(10)62349-5. 21450337 PMC3555696

[5] R. Ma , X. Cai , S. Song , et al., “Association of CVAI With BMD, FRAX Scores, and Osteoporosis Risk in Chinese Elderly Patients With Hypertension,” Scientific Reports 15, no. 1 (2025): 26684, 10.1038/s41598-025-07129-9. 40695842 PMC12284009

[6] S. Song , X. Cai , J. Hu , et al., “Serum Uric Acid and Bone Health in Middle‐Aged and Elderly Hypertensive Patients: A Potential U‐Shaped Association and Implications for Future Fracture Risk,” Metabolites 15, no. 1 (2025): 15, 10.3390/metabo15010015. 39852358 PMC11766991

[7] S. Song , X. Cai , J. Hu , et al., “Effectiveness of Spironolactone in Reducing Osteoporosis and Future Fracture Risk in Middle‐Aged and Elderly Hypertensive Patients,” Drug Design, Development and Therapy 18 (2024): 2215–2225, 10.2147/dddt.S466904. 38882049 PMC11180452

[8] R. K. Srivastava , H. Y. Dar , and P. K. Mishra , “Immunoporosis: Immunology of Osteoporosis‐Role of T Cells,” Frontiers in Immunology 9 (2018): 657, 10.3389/fimmu.2018.00657. 29675022 PMC5895643

[9] G. R. Mundy , “Osteoporosis and Inflammation,” Nutrition Reviews 65, no. 12 Pt 2 (2007): 147–151, 10.1111/j.1753-4887.2007.tb00353.x. 18240539

[10] T. Chen , L. Jin , J. Li , and Y. Liu , “Pyroptosis Mediates Osteoporosis via the Inflammation Immune Microenvironment,” Frontiers in Immunology 15 (2024): 1371463, 10.3389/fimmu.2024.1371463. 38895114 PMC11184911

[11] S. Simsa‐Maziel , J. Zaretsky , A. Reich , Y. Koren , R. Shahar , and E. Monsonego‐Ornan , “IL‐1RI Participates in Normal Growth Plate Development and Bone Modeling,” American Journal of Physiology‐Endocrinology and Metabolism 305, no. 1 (2013): E15–E21, 10.1152/ajpendo.00335.2012. 23592480

[12] K. E. Barbour , R. Boudreau , M. E. Danielson , et al., “Inflammatory Markers and the Risk of Hip Fracture: The Women's Health Initiative,” Journal of Bone and Mineral Research 27, no. 5 (2012): 1167–1176, 10.1002/jbmr.1559. 22392817 PMC3361578

[13] O. C. Kwon , S. Kim , S. Hong , et al., “Role of IL‐32 Gamma on Bone Metabolism in Autoimmune Arthritis,” Immune Network 18, no. 3 (2018): e20, 10.4110/in.2018.18.e20. 29984038 PMC6026691

[14] D. Frase , C. Lee , C. Nachiappan , R. Gupta , and A. Akkouch , “The Inflammatory Contribution of B‐Lymphocytes and Neutrophils in Progression to Osteoporosis,” Cells 12, no. 13 (2023): 1744, 10.3390/cells12131744. 37443778 PMC10340451

[15] J. Ke , F. Qiu , W. Fan , and S. Wei , “Associations of Complete Blood Cell Count‐Derived Inflammatory Biomarkers With Asthma and Mortality in Adults: A Population‐Based Study,” Frontiers in Immunology 14 (2023): 1205687, 10.3389/fimmu.2023.1205687. 37575251 PMC10416440

[16] Y. Xia , C. Xia , L. Wu , Z. Li , H. Li , and J. Zhang , “Systemic Immune Inflammation Index (SII), System Inflammation Response Index (SIRI) and Risk of All‐Cause Mortality and Cardiovascular Mortality: A 20‐Year Follow‐Up Cohort Study of 42,875 US Adults,” Journal of Clinical Medicine Jan 31 12, no. 3 (2023): 1128, 10.3390/jcm12031128. 36769776 PMC9918056

[17] Y. Jiang , B. Luo , W. Lu , et al., “Association Between the Aggregate Index of Systemic Inflammation and Clinical Outcomes in Patients With Acute Myocardial Infarction: A Retrospective Study,” Journal of Inflammation Research 17 (2024): 7057–7067, 10.2147/jir.S481515. 39377046 PMC11457786

[18] Y. Tang , B. Peng , J. Liu , Z. Liu , Y. Xia , and B. Geng , “Systemic Immune‐Inflammation Index and Bone Mineral Density in Postmenopausal Women: A Cross‐Sectional Study of the National Health and Nutrition Examination Survey (NHANES) 2007‐2018,” Frontiers in Immunology 13 (2022): 975400, 10.3389/fimmu.2022.975400. 36159805 PMC9493473

[19] S. Chen , X. Sun , J. Jin , G. Zhou , and Z. Li , “Association Between Inflammatory Markers and Bone Mineral Density: A Cross‐Sectional Study From Nhanes 2007‐2010,” Journal of Orthopaedic Surgery and Research 18, no. 1 (2023): 305, 10.1186/s13018-023-03795-5. 37069682 PMC10108543

[20] G. Davey Smith and G. Hemani , “Mendelian Randomization: Genetic Anchors for Causal Inference in Epidemiological Studies,” Human Molecular Genetics 23, no. R1 (2014): R89–R98, 10.1093/hmg/ddu328. 25064373 PMC4170722

[21] V. W. Skrivankova , R. C. Richmond , B. A. R. Woolf , et al., “Strengthening the Reporting of Observational Studies in Epidemiology Using Mendelian Randomization: The STROBE‐MR Statement,” Journal of the American Medical Association Oct 26 326, no. 16 (2021): 1614–1621, 10.1001/jama.2021.18236. 34698778

[22] R. Xie , M. Xiao , L. Li , et al., “Association Between SII and Hepatic Steatosis and Liver Fibrosis: A Population‐Based Study,” Frontiers in Immunology 13 (2022): 925690, 10.3389/fimmu.2022.925690. 36189280 PMC9520084

[23] S. Huang , W. Xie , Y. Gao , et al., “A Role for Systemic Inflammation in Stroke‐Associated Infection and the Long‐Term Prognosis of Acute Ischemic Stroke: A Mediation Analysis,” Journal of Inflammation Research 17 (2024): 6533–6545, 10.2147/jir.S474344. 39318992 PMC11420892

[24] S. Hosseninia , H. Ghobadi , K. Garjani , S. A. H. Hosseini , and M. R. Aslani , “Aggregate Index of Systemic Inflammation (AISI) in Admission as a Reliable Predictor of Mortality in COPD Patients With Covid‐19,” BMC Pulmonary Medicine 23, no. 1 (2023): 107, 10.1186/s12890-023-02397-5. 37003999 PMC10063934

[25] A. C. Looker , E. S. Orwoll , C. C. Johnston, Jr. , et al., “Prevalence of Low Femoral Bone Density in Older U.S. Adults From Nhanes III,” Journal of Bone and Mineral Research 12, no. 11 (1997): 1761–1768, 10.1359/jbmr.1997.12.11.1761. 9383679

[26] F. Cosman , S. J. de Beur , M. S. LeBoff , et al., “Clinician's Guide to Prevention and Treatment of Osteoporosis,” Osteoporosis International 25, no. 10 (2014): 2359–2381, 10.1007/s00198-014-2794-2. 25182228 PMC4176573

[27] D. Vuckovic , E. L. Bao , P. Akbari , et al., “The Polygenic and Monogenic Basis of Blood Traits and Diseases,” Cell 182, no. 5 (2020): 1214–1231.e11, 10.1016/j.cell.2020.08.008. 32888494 PMC7482360

[28] Z. Lin , Y. Deng , and W. Pan , “Combining the Strengths of Inverse‐Variance Weighting and Egger Regression in Mendelian Randomization Using a Mixture of Regressions Model,” PLoS Genetics 17, no. 11 (2021): e1009922, 10.1371/journal.pgen.1009922. 34793444 PMC8639093

[29] S. Burgess and S. G. Thompson , “Interpreting Findings From Mendelian Randomization Using the MR‐Egger Method,” European Journal of Epidemiology 32, no. 5 (2017): 377–389, 10.1007/s10654-017-0255-x. 28527048 PMC5506233

[30] S. Burgess , J. Bowden , T. Fall , E. Ingelsson , and S. G. Thompson , “Sensitivity Analyses for Robust Causal Inference From Mendelian Randomization Analyses With Multiple Genetic Variants,” Epidemiology 28, no. 1 (2017): 30–42, 10.1097/ede.0000000000000559. 27749700 PMC5133381

[31] Y. Zhang , J. Liang , P. Liu , Q. Wang , L. Liu , and H. Zhao , “The RANK/RANKL/OPG System and Tumor Bone Metastasis: Potential Mechanisms and Therapeutic Strategies,” Frontiers in Endocrinology 13 (2022): 1063815, 10.3389/fendo.2022.1063815. 36589815 PMC9800780

[32] D. Andreev , K. Kachler , M. Liu , et al., “Eosinophils Preserve Bone Homeostasis by Inhibiting Excessive Osteoclast Formation and Activity via Eosinophil Peroxidase,” Nature Communications 15, no. 1 (2024): 1067, 10.1038/s41467-024-45261-8. PMC10844633 38316791

[33] D. Andreev and P. Porschitz , “Emerging Roles of Eosinophils in Bone,” Current Osteoporosis Reports 23, no. 1 (2025): 17, 10.1007/s11914-025-00913-6. 40183859 PMC11971228

[34] P. S. Sharif and M. Abdollahi , “The Role of Platelets in Bone Remodeling,” Inflammation & Allergy Drug Targets 9, no. 5 (2010): 393–399, 10.2174/187152810793938044. 20518723

[35] W. A. Ciovacco , Y. H. Cheng , M. C. Horowitz , and M. A. Kacena , “Immature and Mature Megakaryocytes Enhance Osteoblast Proliferation and Inhibit Osteoclast Formation,” Journal of Cellular Biochemistry 109, no. 4 (2010): 774–781, 10.1002/jcb.22456. 20052670 PMC3095430

[36] C. M. Mikelis , M. Simaan , K. Ando , et al., “RhoA and ROCK Mediate Histamine‐Induced Vascular Leakage and Anaphylactic Shock,” Nature Communications 6, no. 1 (2015): 6725, 10.1038/ncomms7725. PMC4394241 25857352

[37] B. Weicht , P. Maitz , B. Kandler , M. B. Fischer , G. Watzek , and R. Gruber , “Activated Platelets Positively Regulate Rankl‐Mediated Osteoclast Differentiation,” Journal of Cellular Biochemistry. Dec 1 102, no. 5 (2007): 1300–1307, 10.1002/jcb.21360. 17957725

[38] P. Maitz , B. Kandler , M. B. Fischer , G. Watzek , and R. Gruber , “Activated Platelets Retain Their Potential to Induce Osteoclast‐Like Cell Formation in Murine Bone Marrow Cultures,” Platelets. Nov 17, no. 7 (2006): 477–483, 10.1080/09537100600759105. 17074724

[39] M. Scherlinger , C. Richez , G. C. Tsokos , E. Boilard , and P. Blanco , “The Role of Platelets in Immune‐Mediated Inflammatory Diseases,” Nature Reviews Immunology 23, no. 8 (2023): 495–510, 10.1038/s41577-023-00834-4. PMC9882748 36707719

[40] Y. Liu , Y. Zeng , J. Lu , et al., “Correlation of Hemoglobin With Osteoporosis in Elderly Chinese Population: A Cross‐Sectional Study,” Frontiers in Public Health 11 (2023): 1073968, 10.3389/fpubh.2023.1073968. 37124822 PMC10133547

[41] R. Usategui‐Martín , R. Rigual , M. Ruiz‐Mambrilla , J. M. Fernández‐Gómez , A. Dueñas , and J. L. Pérez‐Castrillón , “Molecular Mechanisms Involved in Hypoxia‐Induced Alterations in Bone Remodeling,” International Journal of Molecular Sciences 23, no. 6 (2022): 3233, 10.3390/ijms23063233. 35328654 PMC8953213

[42] D. A. Bemben , V. D. Sherk , S. R. Buchanan , S. Kim , K. Sherk , and M. G. Bemben , “Acute and Chronic Bone Marker and Endocrine Responses to Resistance Exercise With and Without Blood Flow Restriction in Young Men,” Frontiers in Physiology 13 (2022): 837631, 10.3389/fphys.2022.837631. 35370772 PMC8969015

[43] A. Wang , H. Zhang , G. Li , et al., “Deciphering Core Proteins of Osteoporosis With Iron Accumulation by Proteomics in Human Bone,” Frontiers in Endocrinology 13 (2022): 961903, 10.3389/fendo.2022.961903. 36313751 PMC9614156

[44] J. Tsay , Z. Yang , F. P. Ross , et al., “Bone Loss Caused by Iron Overload in a Murine Model: Importance of Oxidative Stress,” Blood. Oct 7 116, no. 14 (2010): 2582–2589, 10.1182/blood-2009-12-260083. 20554970 PMC2953890

[45] J. Luo , L. Li , W. Shi , K. Xu , Y. Shen , and B. Dai , “Oxidative Stress and Inflammation: Roles In Osteoporosis,” Frontiers in Immunology 16 (2025): 1611932, 10.3389/fimmu.2025.1611932. 40873591 PMC12379731

[46] H. Ma , X. Cai , J. Hu , et al., “Association of Systemic Inflammatory Response Index With Bone Mineral Density, Osteoporosis, and Future Fracture Risk in Elderly Hypertensive Patients,” Postgraduate Medicine 136, no. 4 (2024): 406–416, 10.1080/00325481.2024.2354158. 38753519

